# Exergaming as a Functional Test Battery in Patients Who Received Arthroscopic Ankle Arthrodesis: Cross-sectional Pilot Study

**DOI:** 10.2196/21924

**Published:** 2021-05-05

**Authors:** Roel Hendrickx, Tim van der Avoird, Peter Pilot, Gino Kerkhoffs, Martijn Schotanus

**Affiliations:** 1 Zuyderland Medical Centre Sittard Netherlands; 2 ZimmerBiomet Dordrecht Netherlands; 3 Amsterdam University Medical Centers Amsterdam Netherlands

**Keywords:** arthroscopic ankle arthrodesis, exergaming, functional test battery, exergames, serious games, ankle, function, game, exercise, physical activity, rehabilitation, gait, quality of care

## Abstract

**Background:**

Recently, movement-based videogames (exergames) have gained popularity in improving the rehabilitation process after surgery. During exergaming, participants are physically challenged as the game component stimulates adherence to the training program. There is no literature on the effect of exergame training interventions in patients who received arthroscopic ankle arthrodesis.

**Objective:**

This pilot study assessed the potency of an existing exergaming tool for the rehabilitation program of patients who received arthroscopic ankle arthrodesis.

**Methods:**

A cross-sectional pilot study was performed, in which patients who received arthroscopic ankle arthrodesis (n=8) were subjected to an exergaming protocol. Gait analysis was performed with a treadmill system. A healthy age-matched control group (n=10) was used as the control group.

**Results:**

The patient group was capable of performing exergaming exercises and they showed no floor or ceiling effect. Only in case of the overall stability, the patient group performed significantly less better than the control group (*P*=.03). Gait analysis showed equal step length with increased external rotation of the affected limb.

**Conclusions:**

Exergaming seems to be a valuable tool for measuring the ability of patients who received AAA to perform activities of daily living and it has the potential to individualize rehabilitation programs. When exergaming is systematically integrated with patient-reported outcome measures and activity tracking, it has the potential to improve the quality of care.

## Introduction

The use of technology-driven physical activities such as videogames that require participants to be physically active or exercise in order to play the game, also known as exergames, has been proposed as a valuable treatment option to encourage participation in rehabilitation programs and to improve adherence to therapy programs [1]. The challenging element of the game and the accessibility to practice at home will improve the frequency of the exercise, thereby improving the quality and speed of rehabilitation. Further, exergaming might enable patients to participate actively in social and sporting activities sooner [2,3]. Besides using exergaming for rehabilitation, it has the potential to be used for functional assessments, thereby providing more insight into the progress of the patients.

Measuring the ability to perform activities of daily living is very informative for evaluating the recovery of patients. Currently, the functional outcomes in the treatment of musculoskeletal injuries are often evaluated with patient-reported outcome measures (PROMs). However, discrepancies in subjective and objective measures are known, and therefore, easy-to-obtain objective measures are needed [4]. With the use of wearable activity monitors, it is possible to objectively measure the physical activity of patients in a free-living environment. Activity monitors can differentiate between different physical activity types in daily living (eg, stand, walk, sit) [5]. Although the accessibility of activity monitors is very low despite the broad acceptance of smartwatches, detailed information on specific activities of daily living is needed. A potential in the development in this direction is exergaming. During exergaming, a person plays a videogame to perform exercises. Exergaming is used in multiple fields of medicine. Every medical (sub)specialty has its own possibilities and challenges.

Exergaming consists of 2 words, that is, exercise and gaming, and the influence of both aspects during playing needs to be understood. Since this concept is still relatively new, little is known about how big the influence of the gaming part during exergaming is. While playing a regular videogame, the player learns the features of the game in a playful manner. The first level of every game makes the player familiar with the game and the game levels gradually increase in complexity. This poses a challenge for exergaming in rehabilitation. This study focusses on how to determine the influence of the gaming part on the score and progress of the rehabilitation. People who are more acquainted with videogaming may have an advantage and may receive higher scores.

Ankle osteoarthritis is an invalidating condition, which causes pain, dysfunction, and immobility [6]. Osteoarthritis, in general, is treated with nonsteroid drugs and physical therapy. For end-stage osteoarthritis, operative treatment options are available. In case of osteoarthritis of the ankle, total ankle replacement or ankle arthrodesis (AA) are the 2 main surgical solutions. Nowadays, AA is the most practiced treatment for osteoarthritis of the ankle, although there is no consensus in the literature about which treatment is superior [7]. These options mostly lead to significant pain reduction, but they do not offer normal ankle function, thereby leading to long rehabilitation periods to regain the ability to perform activities of daily living. We hypothesized that exergaming could be of additional value for the follow-up of our patients in the near future. This pilot study was a stepwise approach to design a test protocol with an exergaming device for the rehabilitation of patients who received arthroscopic AA (AAA). For extrapolation purposes, we performed a gait analysis with a treadmill system.

## Methods

### Ethical Approval for This Study

This cross-sectional pilot study was performed at the Zuyderland Medical Center, Department of Orthopedic Surgery and Traumatology. This study was approved by the Clinical Research Ethics Committee (protocol 2016/43). Oral and written consent were obtained from all the participants. All patients who received AAA between January 2013 and December 2018 (n=28) at the Zuyderland Medical Center were recruited for study inclusion. Patients were excluded in case of comorbidities with major influence on activities of daily living or when they did not understand the informed consent.

### Participants in This Study

At a median follow-up of 2.5 (IQR 1-5) years after AAA, 35% (8/23) of the patients were able and willing to participate in this study. Causes of ankle arthritis in this study population were posttraumatic arthritis (7/8, 88%) and rheumatoid arthritis (1/8, 13%). The distribution of the affected side was equal (left ankle, 4/8, 50%). A healthy age-matched control group (n=10) was formed, consisting of healthy volunteers, 4 of whom were women (Table 1).

**Table 1:** Characteristics of the patient group and control group.

Characteristics	Patient group (n=8)	Age-matched control group (n=10)	*P* value
Age at participation (years), median (IQR)	66 (55-73)	58 (46-77)	.005^a^
Sex (male), n (%)	6 (75)	6 (60)	.25
Body mass index (kg/m^2^), median (IQR)	28 (27-38)	26 (21-30)	.002^a^

^a^*P* values were calculated by Mann-Whitney *U* test; values were significant at *P*<.05.

Other reasons for not participating in this study were other comorbidities (eg, cerebral vascular accident, heart disease, pulmonary disease), not interested, or not answering the invitation. The test protocol was discussed and designed by subject matter experts (PP, TvdA, and RH). Before the designed protocol was used, it was first tested with a reference group of young healthy men (n=6) recruited from the Department of Orthopedic Surgery and Traumatology. The characteristics and results of this group are described in Multimedia Appendix 1.

### Exergaming Device

**Figure 1:**
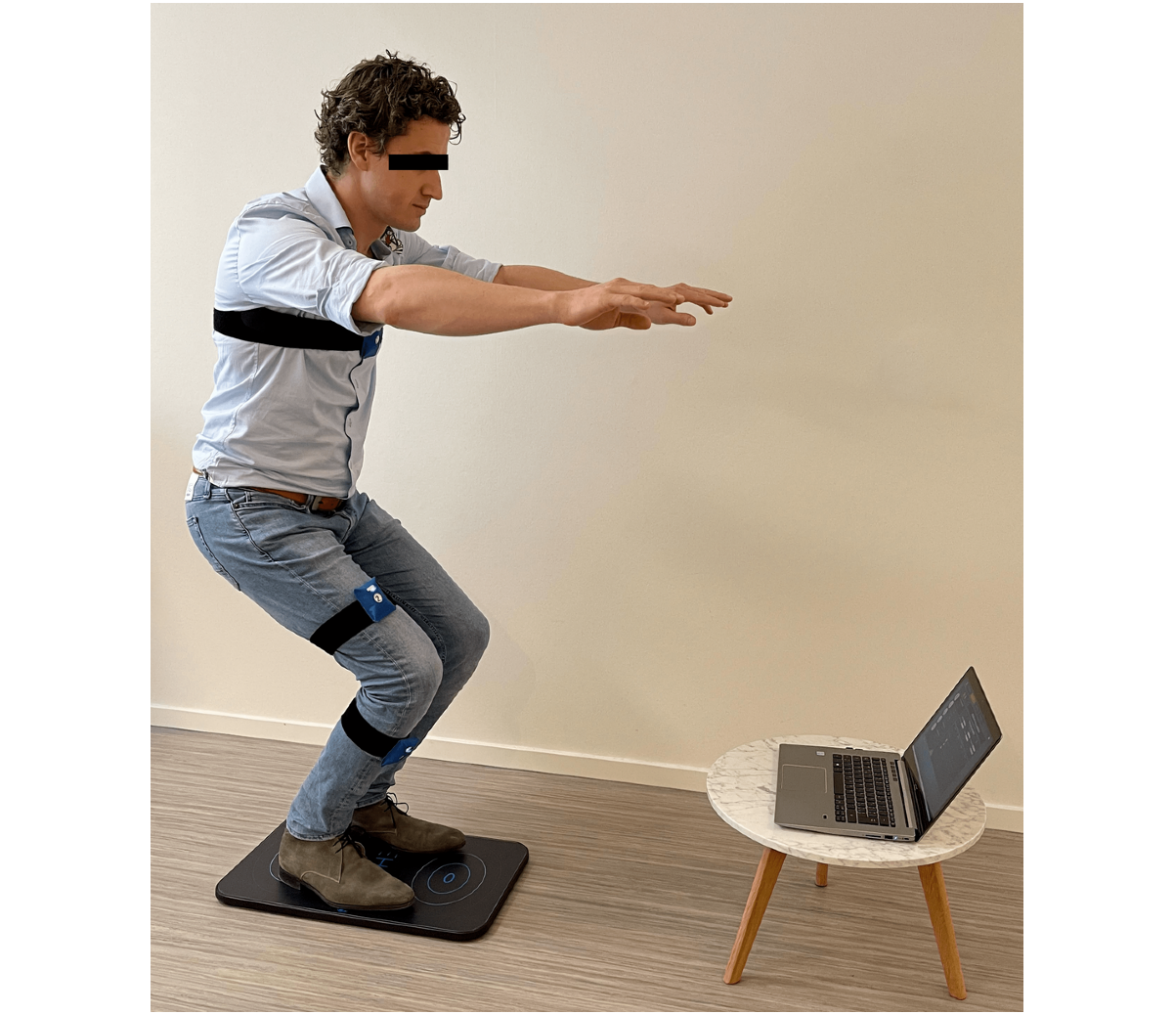
Setup of the Riablo CoRehab system.

The Riablo system (CoRehab s.r.l) includes a laptop with custom Linux SO and Riablo software, 3 inertial Bluetooth sensors (cortex M3 @ 72 MHz, 3D accelerometer [SD 16g], 3D gyroscope [SD 2000 dps], 3D magnetometer [f.s. up to 1 KHz]), Bluetooth pressure board [320 pressure sensors]), and a kit of elastic bands for positioning the sensors (Figure 1). Scores in the report were produced by the Riablo software, which included precision (ability to reach the target angle at the right moment), accuracy (ability not to compensate), and stability (ability to keep balance). The inertial measurement has the necessary accuracy to be safely utilized in rehabilitation programs after orthopedic treatments of the lower limb [8]. This system was used to assess the participant’s functionality. A personal platform was created by entering the details of length, weight, and dominant/injured leg of each patient into the system. This system has a preset library of exercises from which to choose to create work programs suitable for each patient or group of patients. Every exercise needs to be specified with the frequency, intensity, time, and type parameters [9]. As the tool was originally intended as an exergaming tool for sports injuries, a specific protocol had to be developed for exercises that could be done by patients who received ankle arthrodesis during the full period of recovery and that had sufficient discriminative power to give insight into the level of functionality. Exercises that were deemed suitable were weight-mono-lateral transfer, squat, stand and sit, start walking, lunge, reverse lunge, and lateral lunge. Measurements took place in an examination room with a television screen connected to the system. Patients were connected to the system with 3 Bluetooth inertial sensors: one in the middle of the chest and the other two in the middle of the upper leg and middle of the lower leg. Calibration was performed to check the placement and position of the sensors [8]. Before all exercises, a short introduction movie was showed by the system to instruct the patient. Then, the examiner practiced the movement once with the participants. During each exercise, the system gave live feedback to the participant with visual instructions known in games. All patients were granted 2 attempts to perform the exercise correctly; otherwise it was noted as a failure. The best score of each exercise was used. The system produced a report with scores from 0% to 100%, in which a score of 0% was considered to be the lowest score and 100% as the highest score. The quality was registered with indexes that show how each exercise was performed from a quality perspective. The score was built from 3 aspects by assessing the patient for precision (ability to reach the right target at the right time), patient stability during exercise (ability to maintain balance and limit the thorax sways), and accuracy (ability to avoid compensations).

### Zebris FDM-T

**Figure 2:**
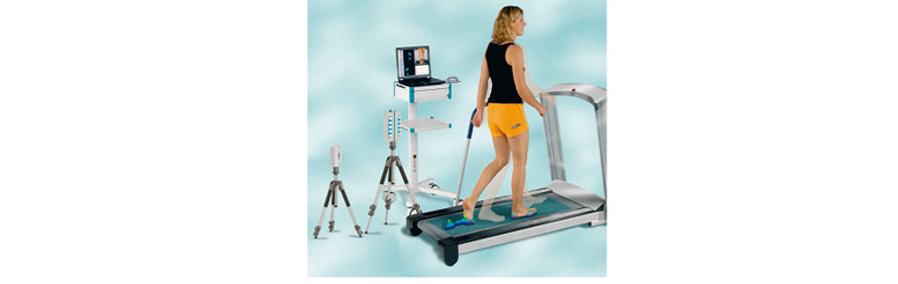
Setup of the Zebris FDM-T treadmill.

The Zebris FDM-T (Figure 2) includes a laptop with Zebris FDM software Suite, treadmill Zebris-FDM Maxxus, and high-speed SYNCLight Cam (100 Hz, pressure plate FDM 1.5 [158×60.5×2.1 cm], sensor area [149×54.2 cm], 11.264 sensors, sampling rate: 100 Hz, optional 200 Hz/300 Hz, measuring range, 1-20 N/cm). Participants were asked to stand in the middle of the treadmill for 10 seconds to measure the static plantar pressure. Gait analysis was obtained with participants walking barefoot for 1 minute on the treadmill at a pace that was comfortable for a short amount of time. Finally, participants were asked to walk with their shoes on the treadmill for 1 minute at the same pace. In the analysis, the following parameters were examined: step length (cm) and external foot rotation (degrees). This system is validated for measuring the spatiotemporal parameters of gait [10].

### Statistical Analysis

Nonparametric statistics were used due to the small sample size. Descriptive statistics were calculated by Mann-Whitney *U* test (continuous variables) to compare the medians between the different groups and Fisher exact test (binary variables) to compare the medians (range). The number of participants were undersized for each group; therefore, the Kolmogorov-Smirnov test was used to compare the results of the CoRehab and Zebris between both groups. The Kolmogorov-Smirnov test was used to compare the medians of different categories between the groups. In this study, the median would produce more representative results instead of the mean. The statistical significance level was comparable or smaller than .05. The data were analyzed using SPSS (version 25.0, IBM Corporation).

## Results

No significant differences were observed between the physical functioning of the patient group and the control group (Table 2). The AAA group had a median overall score of 51% (IQR 45%-69%), which was measured with the exergaming system, whereas the control group had a median overall score of 60% (IQR 28%-79%). In the AAA group, the best performed exercise was the reverse lunge with a median score of 78% (IQR 60%-89%) and the worst performed exercise was the squat with a median score of 11% (IQR 0%-42%). The best performed exercises in the control group were the start to walk (median 79% [IQR 43%-90%]) and the lateral lunge (median 79% [38%-91%]) and the worst performed exercise was the squat (median 12% [IQR 0%-73%]).

**Table 2:** Results of the exercise protocol per group.

Exercises	Patient group score (%), median (IQR)	Age-matched control group score (%), median (IQR)	*P* value
Weight-mono-lateral transfer	50 (0-59)	67 (11-86)	.07
Squat	11 (0-42)	12 (0-73)	.32
Stand and sit	62 (28-77)	68 (23-91)	.49
Start walking	75 (43-90)	79 (43-90)	.52
Lunge	65 (45-79)	52 (4-85)	.20
Reverse lunge	78 (60-89)	74 (8-91)	.21
Lateral lunge	69 (38-91)	79 (28-93)	.61
Overall score	51 (45-69)	60 (28-79)	.32
Average precision	58 (49-75)	65 (35-84)	.34
Average accuracy	87 (67-93)	89 (84-95)	.17
Average stability	73 (59-79)	80 (68-90)	.03^a^

^a^*P* values were calculated by Mann-Whitney *U* test, and they were significant at *P*<.05.

Gait analysis showed more external rotation on the operated site compared to the nonoperated site. The results of the gait analyses are shown in Table 3. The software determines foot rotation as the angle formed by the midline of the foot and the midline of the treadmill. A skewed gait resulting in erroneous values was assessed with camera views and consequently disregarded for further evaluation.

**Table 3:** Descriptive results of the gait analysis per group for step length and rotation.

Gait analysis	Patient group^a^ (n=7)		Age-matched control group^b^ (n=7)		
	Operated ankle	Nonoperated ankle	Right ankle	Left ankle	
Step length (cm), median (IQR)	50.0 (33 to 64)	49.0 (33 to 64)	46 (38 to 47)	44 (39 to 47)	
Rotation (degrees), median (IQR)	9.3 (–2.6 to 13.9)	3.2 (0.9 to 7.6)	13.9 (9.10 to 22.5)	10.1 (1.9 to 21.8)	

^a^Incorrect measurements were obtained from 1 participant in this group.

^b^Incorrect measurements were obtained from 3 participants in this group.

## Discussion

In this study, with exergaming, the patients who received AAA had a median overall score of 51% (IQR 45%-69%), thus indicating sufficient potential for showing improvement but also having the possibility to indicate deterioration at a median follow-up of 2.5 (IQR 1-5) years after receiving AAA. All patients liked the concept of testing. The big challenge in exergaming is to estimate what the limitations caused by the AAA on the practice effect are and how the score is limited to the skills effect of the gaming. Within this challenge, we have the learning effect of this type of exercise in general, and we have the difficulties for every individual exercise. To counter the learning effect, each participant was granted 2 attempts per individual exercise. In several cases, the participants reported that they found the gaming part during the exercise inspiring, but they sometimes missed the experience and the finesse to do the game. Every individual exercise had its own specific characteristics and patients responded quite heterogeneously to this, but with sufficient room to improve or worsen, as depicted in the average scores. During conventional rehabilitation, these characteristics are usually adjusted during the therapy. In exergaming, these have to be defined beforehand, considering the special skills of the therapist, but the outcome scores need to be considered with more objectivity.

For many rehabilitation protocols finding the right exercises and setting, the parameters are the biggest challenge. The parameters of the exercise components, that is, frequency, intensity, time, and type, as described by Knols et al [9], together form a set of guidelines that help to set up exercise routines. These guidelines should fit the exercise goals and the trainee’s level of fitness and is one of the foundations of successful exercise interventions. This combined with the large variance in gaming skills makes it a challenge to find the right testing and training protocol since little experience exists. To obtain a relevant set of exercises, we went through several stages. The available exercises were evaluated whether they would fit the anticipated limitations due to arthrodesis. This process of adapting an exergame for a specific pathology is believed to add positive value, but it also requires experience on both exergaming and the specific pathology [11].

Functional deficit following AA is obvious when comparing to the function of the normal population. Nevertheless, good functional outcomes have been reported mainly using PROMs as an outcome measure [12]. Schuh et al [13] described that most people with ankle osteoarthritis perform activities such as cycling, swimming, hiking, and skiing both at end-stage osteoarthritis and after AA. PROMs are currently used to quantify a disease state or an interventional outcome as perceived by the patient [14]. PROMs suffer from their subjective nature, recall bias, being a time-consuming methodology, low response rates, and completion rate or transcription errors [15]. Furthermore, various PROMs do not capture the changes due to a lack of power of the scores as averse to a lack of change (eg, floor and ceiling effects) [16]. Ankle osteoarthritis results in an unnatural gait pattern. Therefore, gait analysis is done in many AAA studies. Deleu et al [17] quantified the alterations in gait in their meta-analysis. They observed an increase in the walking speed, while step length remained constant. In this study, negative foot rotation angles were found. This can be explained by the angle between the longitudinal axis of the foot and the walking direction; negative values indicate an inward rotation and positive values indicate an outward rotation. Healthy human walking is symmetrical and economical; however, the walking of people who received AA is often asymmetrical and requires more energy [18].

In this study, a mobile treadmill was used to assess the gait pattern at the final follow-up. Our findings were consistent with those of Deleu et al [17]. The increased external rotation during walking compensates for the diminished movement in the ankle. It shortens the lever arm of the forefoot in the sagittal plane, thereby aiding in shifting the weight from back to front [19]. As our population exhibits a walking pattern that resembles that reported by Deleu et al [17], we may assume that our sample represents the “normal” ankle arthrodesis population. Exergaming has some interesting features that may assist in offering high value rehabilitation monitoring while minimizing outpatient controls. The “exer” part enables health care providers to develop pathology/patient-specific routines that change during the rehabilitation process. If exergaming is introduced before surgery, there is ample time to get acquainted with the gaming part and eliminate the confounding factors, as most as possible, thereby emphasizing the advantages of exergaming. The “gaming” part has 2 important strengths. First, it adds a fun factor, which might help in improving adherence to a rehabilitation program [2,3]. Second, it reflects the performance level. As specific goals are achieved, the game will automatically evolve to the next level in which the exercises will drive the rehabilitation process forward.

With the gaining popularity of smartwatches, the amount of research assessing their ability to track mobility and activity is expanding [20]. Shofer et al [21] concluded that a major positive change was seen at 6 months following ankle arthrodesis. Although step activity demonstrated no improvement at 6 months following ankle arthrodesis, the total number of steps as well as the high-frequency steps continued to improve significantly for up to 3 years following surgery. Exergaming could add the more qualitative assessment of movement to this activity part.

This study has a few limitations. We describe a small population; therefore, one needs to be careful with extrapolation. However, this pilot study supports future studies in using exergaming for monitoring the rehabilitation process. This study offers sound insights to give direction to future work. In the near future, we think that the findings of our study might be helpful in creating a platform for high-quality rehabilitation that is largely home-based with continuous distant monitoring and feedback. The specific goals during different phases of rehabilitation need further attention. We believe that the first phase after immobilization should have a different focus with a specific set of exercises. In case of limited swelling and pain, the final phase should have exercises focusing on the desired endpoint.

## Appendix

Multimedia Appendix 1 Characteristics and results of the healthy reference group.

## Abbreviations

AA: ankle arthrodesis
AAA: arthroscopic ankle arthrodesis
PROM: patient-reported outcome measure

## References

[1] Tobler-Ammann BC, Surer E, Knols RH, Borghese NA, de Bruin ED (2017). User Perspectives on Exergames Designed to Explore the Hemineglected Space for Stroke Patients With Visuospatial Neglect: Usability Study. JMIR Serious Games.

[2] Yong Joo Loh, Soon Yin Tjan, Xu D, Thia E, Pei Fen Chia, Kuah CWK, Kong K (2010). A feasibility study using interactive commercial off-the-shelf computer gaming in upper limb rehabilitation in patients after stroke. J Rehabil Med.

[3] Jorgensen MG, Laessoe U, Hendriksen C, Nielsen OBF, Aagaard P (2013). Efficacy of Nintendo Wii training on mechanical leg muscle function and postural balance in community-dwelling older adults: a randomized controlled trial. J Gerontol A Biol Sci Med Sci.

[4] Luna IE, Kehlet H, Peterson B, Wede HR, Hoevsgaard SJ, Aasvang EK (2017). Early patient-reported outcomes objective function after total hip and knee arthroplasty: a prospective cohort study. Bone Joint J.

[5] Jelsma J, Schotanus MGM, Buil ITAF, van Kuijk SMJ, Heyligers IC, Grimm B (2020). Patients with hip resurfacing arthroplasty are not physically more active than those with a stemmed total hip. Acta Orthop.

[6] Saltzman CL, Salamon ML, Blanchard GM, Huff T, Hayes A, Buckwalter JA, Amendola A (2005). Epidemiology of ankle arthritis: report of a consecutive series of 639 patients from a tertiary orthopaedic center. Iowa Orthop J.

[7] Lawton CD, Butler BA, Dekker RG, Prescott A, Kadakia AR (2017). Total ankle arthroplasty versus ankle arthrodesis-a comparison of outcomes over the last decade. J Orthop Surg Res.

[8] Leardini A, Lullini G, Giannini S, Berti L, Ortolani M, Caravaggi P (2014). Validation of the angular measurements of a new inertial-measurement-unit based rehabilitation system: comparison with state-of-the-art gait analysis. J Neuroeng Rehabil.

[9] Knols RH, Vanderhenst T, Verra ML, de Bruin ED (2016). Exergames for Patients in Acute Care Settings: Systematic Review of the Reporting of Methodological Quality, FITT Components, and Program Intervention Details. Games Health J.

[10] Reed LF, Urry SR, Wearing SC (2013). Reliability of spatiotemporal and kinetic gait parameters determined by a new instrumented treadmill system. BMC Musculoskelet Disord.

[11] Skjæret Nina, Nawaz A, Morat T, Schoene D, Helbostad JL, Vereijken B (2016). Exercise and rehabilitation delivered through exergames in older adults: An integrative review of technologies, safety and efficacy. Int J Med Inform.

[12] Hendrickx RPM, Stufkens SAS, de Bruijn EE, Sierevelt IN, van Dijk CN, Kerkhoffs GMMJ (2011). Medium- to long-term outcome of ankle arthrodesis. Foot Ankle Int.

[13] Schuh R, Hofstaetter J, Krismer M, Bevoni R, Windhager R, Trnka H (2012). Total ankle arthroplasty versus ankle arthrodesis. Comparison of sports, recreational activities and functional outcome. Int Orthop.

[14] Rolfson O, Eresian Chenok K, Bohm E, Lübbeke Anne, Denissen G, Dunn J, Lyman S, Franklin P, Dunbar M, Overgaard S, Garellick G, Dawson J, Patient-Reported Outcome Measures Working Group of the International Society of Arthroplasty Registries (2016). Patient-reported outcome measures in arthroplasty registries. Acta Orthop.

[15] Pronk Y, Pilot P, Brinkman JM, van Heerwaarden RJ, van der Weegen W (2019). Response rate and costs for automated patient-reported outcomes collection alone compared to combined automated and manual collection. J Patient Rep Outcomes.

[16] Weber BA, Yarandi H, Rowe MA, Weber JP (2005). A comparison study: paper-based versus web-based data collection and management. Appl Nurs Res.

[17] Deleu P, Besse J, Naaim A, Leemrijse T, Birch I, Devos Bevernage B, Chèze Laurence (2020). Change in gait biomechanics after total ankle replacement and ankle arthrodesis: a systematic review and meta-analysis. Clin Biomech (Bristol, Avon).

[18] Stenum J, Choi JT (2020). Step time asymmetry but not step length asymmetry is adapted to optimize energy cost of split-belt treadmill walking. J Physiol.

[19] Hefti FL, Baumann JU, Morscher EW (1980). Ankle joint fusion -- determination of optimal position by gait analysis. Arch Orthop Trauma Surg.

[20] Small SR, Bullock GS, Khalid S, Barker K, Trivella M, Price AJ (2019). Current clinical utilisation of wearable motion sensors for the assessment of outcome following knee arthroplasty: a scoping review. BMJ Open.

[21] Shofer JB, Ledoux WR, Orendurff MS, Hansen ST, Davitt J, Anderson JG, Bohay D, Coetzee JC, Houghton M, Norvell DC, Sangeorzan BJ (2019). Step Activity After Surgical Treatment of Ankle Arthritis. J Bone Joint Surg Am.

