# Supplementary material for: Exergaming as a Functional Test Battery in Patients Who Received Arthroscopic Ankle Arthrodesis: Cross-sectional Pilot Study
Source: JMIR Rehabil Assist Technol. 2021 May 5;8(2):e21924. doi: 10.2196/21924 (PMC8135032; doi:10.2196/21924)
Supplement: Multimedia Appendix 1 [file rehab_v8i2e21924_app1.docx]

Table S1. Characteristics of young reference group (RG).

| **Patient characteristics** | **Reference group (RG)**  Median (range)  N=6 |
| --- | --- |
| **Age at participation study , years** | 25 (22 - 27) |
| **Sex, female/male** | 0/6 |
| **Injured side, left/right** | - |
| **Body mass index, kg/m^2^** | 23 (20 - 31) |
| **Rheumatoid arthritis** | - |
| **Posttraumatic osteoarthritis** | - |

Table S2. Results for the different exercises.

| **Exercises, score in %** | **Reference group (RG)**  Median (range)  N=6 |
| --- | --- |
| **Weight-mon-lateral transfer** | 71 (59 - 85) |
| **Squat** | 50 (32 - 71) |
| **Stand and sit** | 85 (78 - 94) |
| **Start walking** | 79 (74 - 88) |
| **Lunge** | 90 (77 - 98) |
| **Reverse lunge** | 95 (79 - 97) |
| **Lateral lunge** | 94 (92 - 97) |
| **Overall score** | 79 (76 - 86) |
| **Adjusted overall score**** | 85 (79 - 92) |
| **Ave. Precision** | 84 (82 - 91) |
| **Ave. Accuracy** | 92 (81 - 94) |
| **Ave. Stability** | 80 (71 - 82) |

Table S3. Gait analysis for the RG.

| **Gait analysis** | **Reference group (RG)**  Median (range) N= 6 (range) |
| --- | --- |
| **Step length right foot (cm)** | 61 (49 - 68) |
| **Step length left foot (cm)** | 58 (51 - 67) |
| **Rotation right foot (degrees)** | 9.1 (0.6 - 15.1) |
| **Rotation left foot (degrees)** | 6.0 (-0.1 - 15) |
